# Implication of *Anopheles funestus* in malaria transmission in the city of Yaoundé, Cameroon

**DOI:** 10.1051/parasite/2020005

**Published:** 2020-02-12

**Authors:** Landre Djamouko-Djonkam, Diane Leslie Nkahe, Edmond Kopya, Abdou Talipouo, Carmene Sandra Ngadjeu, Patricia Doumbe-Belisse, Roland Bamou, Parfait Awono-Ambene, Timoléon Tchuinkam, Charles Sinclair Wondji, Christophe Antonio-Nkondjio

**Affiliations:** 1 Laboratoire de Recherche sur le Paludisme, Organisation de Coordination Pour la Lutte Contre les Endémies en Afrique Centrale (OCEAC) P.O. Box 288 Yaoundé Cameroon; 2 Vector Borne Diseases Laboratory of the Applied Biology and Ecology Research Unit (VBID-URBEA), Department of Animal Biology, Faculty of Sciences of the University of Dschang P.O. Box 067 Dschang Cameroon; 3 Faculty of Science, University of Yaoundé I P.O. Box 337 Yaoundé Cameroon; 4 Vector Biology, Liverpool School of Tropical Medicine Pembroke Place L3 5QA Liverpool UK

**Keywords:** Malaria, Transmission, *Anopheles funestus*, *Anopheles gambiae*, Urban, Yaoundé, Cameroon

## Abstract

The contribution of *Anopheles funestus* to malaria transmission in the urban environment is still not well documented. The present study assesses the implication of *An. funestus* in malaria transmission in two districts, Nsam and Mendong, in the city of Yaoundé. Adult mosquitoes were collected using Centers for Disease Control and Prevention miniature light traps (CDC-LT) and human landing catches from April 2017 to March 2018 and were identified morphologically to the species level. Those belonging to the *Anopheles gambiae* complex and to the *Anopheles funestus* group were further processed by PCR to identify members of each complex/group. Anopheline mosquitoes were analysed to determine their infection status using an enzyme-linked immunosorbent assay. Bioassays were conducted with 2–5-day-old female *Anopheles funestus* and *An. gambiae* s.l. to determine their susceptibility to permethrin, deltamethrin and dichlorodiphenyltrichloroethane (DDT). Six anopheline species were collected in the peri-urban district of Mendong: *Anopheles gambiae*, *An. coluzzii*, *An. funestus*, *An. leesoni*, *An. ziemanni* and *An. marshallii*; only four out of the six were recorded in Nsam. Of the two members of the *Anopheles gambiae* complex collected, *An. coluzzii* was the most prevalent. *Anopheles coluzzii* was the most abundant species in Nsam, while *An. funestus* was the most abundant in Mendong. Both *Anopheles funestus* and *An. gambiae* s.l. were found to be infected with human *Plasmodium* at both sites, and both were found to be resistant to DDT, permethrin, and deltamethrin. This study confirms the participation of *An. funestus* in malaria transmission in Yaoundé and highlights the need to also target this species for sustainable control of malaria transmission.

## Abstract (fr)

La contribution d’*Anopheles funestus* à la transmission du paludisme en milieu urbain n’est pas encore bien documentée. La présente étude évalue l’implication d’*An. funestus* dans la transmission du paludisme dans la ville de Yaoundé. L’étude a été menée dans deux quartiers de la ville de Yaoundé, Nsam et Mendong. Des moustiques adultes ont été collectés à l’aide des pièges lumineux de type CDC et sur volontaires humains d’avril 2017 à mars 2018. Les moustiques appartenant au genre *Anopheles* ont été analysés afin de déterminer leur statut infectant grâce à la technique ELISA. Des bio-essais ont été effectués sur des femelles d’*An. funestus* et *An. gambiae* s.l. âgées de 2 à 5 jours afin de déterminer leur sensibilité à la perméthrine, la deltaméthrine et le dichlorodiphényltrichloroéthane (DDT). Six espèces d’anophèles ont été collectées dans le quartier périurbain de Mendong : *An. gambiae*, *An. coluzzii*, *An. funestus*, *An. leesoni*, *An. ziemanni* et *An. marshallii* ; seule les quatre premières ont été trouvées à Nsam. Des deux espèces du complexe *An. gambiae* retrouvées, *An. coluzzii* était la plus abondante. *Anopheles gambiae* s.l. était l’espèce prédominante à Nsam, tandis que *An. funestus* était la plus abondante à Mendong. Les deux espèces *An. funestus* et *An. gambiae* s.l. étaient infectées par des *Plasmodium* humains dans les deux sites, et se sont avérés résistantes au DDT, à la perméthrine et à la deltaméthrine. Cette étude confirme la participation d’*An. funestus* à la transmission du paludisme à Yaoundé et souligne la nécessité de cibler également cette espèce pour une lutte durable contre le paludisme.

## Introduction

Anthropogenic changes affecting the environment, such as large-scale unplanned urbanisation, are considered to have a major influence on vector-borne disease epidemiology [27, 29, 31]. In Cameroon, the city of Yaoundé is one of the largest in the country with a population of approximately three million inhabitants, and it has experienced intense modification of its natural environment throughout the past years. The rapid and spontaneous urbanisation in and around the city centre, with the absence of infrastructure for sanitation and surface water drainage, as well as the colonisation of lowland areas for urban agriculture or housing construction, has favoured the establishment of anophelines in an urban setting [5, 21]. In Cameroon, malaria is still a major public health problem affecting approximately 30% of the population annually [51, 64], and its prevention relies entirely on the use of insecticide-treated nets (ITNs) and/or long-lasting insecticidal nets (LLINs) [51]. However, overreliance on insecticides for both public health and agriculture over the past decades has contributed to the emergence and rapid expansion of pyrethroid resistance particularly in urban settings [5, 8, 9, 49]. In Yaoundé, *Anopheles gambiae* and *An. coluzzii* are the most important malaria vectors in the city centre [56, 66]. Studies conducted at the periphery of the city of Yaoundé reported the presence of vectors such as *An. moucheti*, *An. nili*, and *An. funestus,* which contribute alongside *An. gambiae* s.l. to malaria transmission [2]. *Anopheles funestus* has always been reported in sympatry with *An. gambiae* in most rural settings in Cameroon [7, 10, 18]. In some places, this vector was found to perpetuate high infection rates surpassing those of *An. gambiae*, demonstrating the important epidemiological role that it can play [16]. *Anopheles funestus* has a close relationship to humans and usually displays high anthropophilic and endophilic behaviour [11, 20]. Similar to *An. gambiae*, high insecticide resistance has been reported for this vector population in Cameroon [38]. Resistant populations were found to overexpress different detoxification genes conferring resistance to organochlorines and pyrethroids [33, 39, 47]. The following examples highlight the increasing challenges for controlling these vector populations. To date, three species belonging to the *An. funestus* species group have been reported in Cameroon, including *An. funestus*, *An. leesoni*, and *An. rivulorum* [15]; however, only *An. funestus* is largely distributed across the country and has a major role in malaria transmission [2, 3, 11, 15]. Despite being recognised as a major malaria vector in Africa, the epidemiological role of *An. funestus* in the urban environment has only been explored with limited scope in both Cameroon and across Africa [14]. In the present study, the role of *An. funestus* in malaria transmission in the city of Yaoundé was investigated during a survey comparing malaria transmission dynamics between a central and a peri-urban district.

## Methods

### Ethics approval and consent to participate

The study was conducted under the ethical clearance No. 2016/11/832/CE/CNERSH/SP conferred by the Cameroon National Ethics Committee for Research on Human Health (CNERSH) Ref. No. D30-172/L/MINSANTE/SG/DROS/TMC on 4 April 2017. For human landing catches, all adult men who took part in the collection signed a written informed consent form before being enrolled in the study, as recommended by the validated protocol and were given free malaria prophylaxis.

### Study area

The study was conducted in Yaoundé (3°43′00″–3°58′00″ N and 11°24′30″–11°34′30″ E), the capital city of Cameroon. Yaoundé is a city covering a surface area of 304 km^2^ and situated 700 m above sea level. Its population is estimated to be approximately three million inhabitants. The city is drained by several permanent streams and is situated within the Congo-Guinean phytogeographic zone, characterised by vegetation dominated by Sterculiaceae and Ulmaceae [35]. The climate is of the equatorial type and comprises two rainy seasons extending from March to June and from September to November. The average rainfall in Yaoundé is estimated to be 1688 mm/year with an average annual temperature of 26 °C. The average humidity is 80% and varies during the day between 35% and 98% [57]. The city is exposed to frequent humid winds blowing south-west to west or north to west [62].

**Figure 1:**
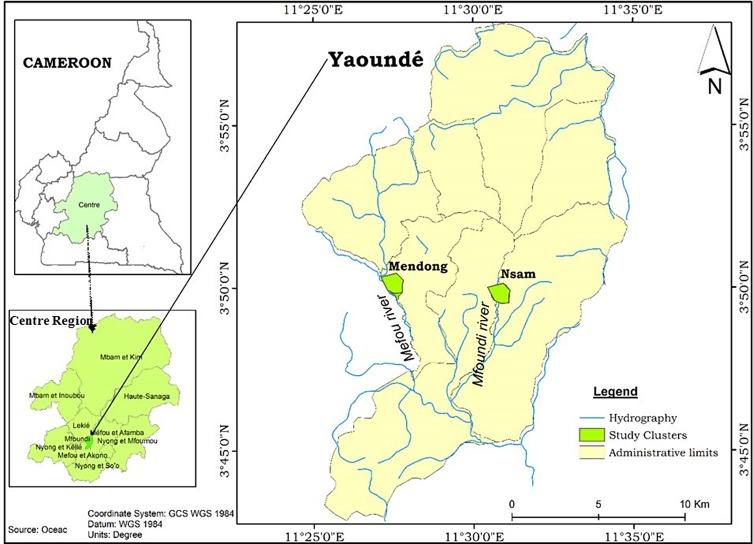
A map of Yaoundé showing the study sites of Mendong and Nsam.

Mosquito collections were conducted in the districts of Nsam and Mendong (Fig. 1). Nsam is situated in the city centre along the Mfoundi river, which provides excellent breeding opportunities for mosquitoes during the dry season. During the rainy season, breeding habitats are evenly distributed in the district; however, most of them are found in lowland areas. Mendong is a district situated at the periphery of the city along the Mefou river. The district is highly populated with construction in both highland and lowland areas (Table 1). Marshlands along the Mefou river are exploited for house construction and for the practice of market gardening during the dry season. The emergent vegetation in swamps and along the edges of the Mefou river provides ideal breeding opportunities for *An. funestus*, the predominant anopheline species in the district. The practice of agriculture in marshland during the dry season also promotes the presence of this species all year long.

**Table 1:** Characteristics of the study sites in Nsam and Mendong districts.

Characteristic	Nsam	Mendong
Situation	City centre	City periphery
Estimated population	20,000	30,000
Practice of urban agriculture	No	Yes
Presence of temporary breeding sites	++	+++
Presence of permanent breeding sites	+++	++++
LLIN ownership	96%	95%
Presence of cattle	No	Yes
Presence of other domestic animals (chickens, pigs, goats, sheep, etc.)	Yes	Yes
Rearing of fish in pools	Yes	Yes
Rivers	Mfoundi	Mefou

++: low; +++: high; ++++: very high.

### Adult mosquito collection

Two collection methods targeting host-seeking mosquitoes – Centers for Disease Control and Prevention miniature light traps (CDC-LT) (Model 512 6VDC John W. Hock) and human landing catches (HLCs) – were used from 07:00 pm to 06:00 am indoors and outdoors. Collections were undertaken during the months of April, June, August, October, and December in 2017 and March 2018. Collections using CDC light traps were conducted using 17–20 traps placed indoors and outdoors in a maximum of 12 different houses. In five houses, traps were placed both in and outdoors, whereas in the remaining houses, traps were placed exclusively indoors. Houses were chosen randomly and were separated from one another by a distance of approximately 100 m. Mosquito collections were undertaken in each district over three consecutive nights each month to control for bias such as variation from rainfall and temperature (Table 2). CDC-LTs (Model 512 6VDC John W. Hock) were used as the main method to assess mosquito dynamics.

**Table 2:** Monthly average temperature and precipitation/rainfall of 2017 in Yaoundé.^a^

	January	February	March	April	May	June	July	August	September	October	November	December
Average *T* (°C)	26.5	27	27.5	27	25.5	25.5	24	23.5	24	24	25	26.5
Min *T* (°C)	20	20	21	21	20	21	20	20	20	20	20	21
Max *T* (°C)	33	34	34	33	31	30	28	27	28	28	30	32
Precipitation (mm)	21	15	46	64	75	32	30	85	134	165	100	19

a https://www.historique-meteo.net/afrique/cameroun/yaounde/2017/

T: temperature.

For human landing catches, collections were conducted in three houses (different from those used for CDC-LTs) in each district, both indoors and outdoors for one night. This collection technique was used to check the efficiency of CDC-LT collections. Once collected, mosquitoes were put into separate bags, and labelled according to the site, night and hour of collection. Local field collectors conducted catches from 07:00 pm to 06:00 am. After giving their consent, they were followed throughout the study and were treated using an artemisinin-based combination if they were detected as having malaria, as recommended by the Cameroon Ministry of Health.

### Mosquito processing

Once collected, anophelines were separated from culicines using the morphological identification keys developed by Edwards [22]. Different anopheline species were also identified using morphological identification keys [25, 26]. Each anopheline specimen was stored individually in a numbered tube containing desiccant, archived and kept in a freezer at −20 °C.

Mosquitoes belonging to the *An. gambiae* complex were further processed by PCR [55] to distinguish *An. coluzzii* from *An. gambiae*, the two members of the complex found in Yaoundé. Molecular identification of members of the *An. funestus* group was conducted using the protocol developed by Koekemoer et al. [32]. DNA extracted from the wings and legs of the mosquitoes, according to the protocol developed by Livak and Schmittgen [36] was used for these analyses.

The head and thorax of female anophelines were tested for the presence of circumsporozoite protein (CSP) from *Plasmodium falciparum* using an enzyme-linked immunosorbent assay (ELISA), as described in Fontenille et al. [24].

### Susceptibility to insecticides

Susceptibility of *An. gambiae* and *An. funestus* to DDT (4%), permethrin (0.75%) and deltamethrin (0.05%) was assessed using the WHO protocol [65]. Wild females of *Anopheles funestus* collected using HLC were kept alive to lay eggs. Larvae obtained from the eggs were reared to adults and used for bioassays. Wild females were identified to the species level. For *Anopheles gambiae*, larvae collected in temporary water collection in the field were reared to adults. Sampling was conducted from April to May 2017 during the short rainy season. Unfed Females of both species aged 2–5 days old were used to perform insecticide susceptibility tests. Batches of 20–25 mosquitoes per tube were exposed to impregnated papers for 1 h. The number of mosquitoes knocked down by the insecticide was recorded every 10 min during exposure. After exposure, mosquitoes were fed with a 10% glucose solution, and the number of dead mosquitoes was recorded 24 h post-exposure. Tests using untreated papers were conducted as controls. The mortality rates were corrected using the Abbot formula [1] whenever the mortality rate of the controls was between 5% and 20%. Susceptibility and resistance levels were assessed according to the World Health Organization criteria [63]. Female anophelines at the end of the tests were classified into three groups: insecticide resistant (if the mortality rate was <80%), insecticide tolerant (if the mortality rate was between 80% and 97%), and insecticide susceptible (if the mortality rate was >97%).

To detect the presence of the *kdr* alleles (L1014F and L1014S) conferring resistance to DDT and pyrethroids, DNA extracted from the wings and legs of a sub-sample of *An. gambiae* s.l. females was screened using a TaqMan assay [12]. The DNA was also used for species identification.

### Data analysis

The biting rate (number of bites per person per night) was calculated as the number of mosquitoes caught in one night divided by the number of collectors. The infection rate was calculated as the number of infected anophelines divided by the total number processed. The entomological inoculation rate (EIR) was calculated by multiplying the infection rate by the human biting rate for one night, to obtain the daily EIR. The EIR for the CDC light traps was estimated as follows: EIR = 1.605 × (No. of sporozoite positive ELISAs/No. of mosquitoes tested) × (No. of mosquitoes collected/No. of CDC light traps). The 1.605 represents the factor of overestimation of human landing catches compared to light traps. The monthly EIR was calculated by multiplying the average daily EIR by the number of days in the month. The annual EIR was calculated by summing the monthly EIR for a year. The confidence interval was calculated using MedCalc statistical software, version 15.8 (MedCalc software bvba, Ostend, Belgium; https://www.medCalc.org; 2015). Statistical analyses were performed with SPSS Statistics for Windows, version 20 (SPSS Inc., Chicago, IL, USA) to compare percentages or averages. The level of significance of each test was set at *p* = 0.05.

## Results

### Species diversity

A total of 7136 mosquitoes belonging to four genera were collected in both Mendong and Nsam. Of these, 5160 were collected in Nsam and 1976 in Mendong. Mosquitoes collected included *Culex*, *Anopheles*, *Mansonia*, and *Aedes* species at both sites. *Culex* species were the most abundantly represented with 88.1% and 58.7% of the total mosquitoes collected in Nsam and Mendong, respectively (Table 3). High anopheline species diversity was recorded in the peri-urban district of Mendong with six species collected, *An. gambiae, An. coluzzii, An. funestus, An. leesoni, An. ziemanni*, and *An. marshallii*, whereas only four were recorded in the urban district of Nsam (*An. gambiae, An. coluzzii, An. funestus, An. leesoni). Anopheles gambiae* emerged as the most abundant anopheline species in Nsam (10.3% of the total), while *An. funestus* was the most abundant in Mendong (19% of the total).

**Table 3:** Mosquito species composition in Nsam and Mendong, Yaoundé, from April 2017 to March 2018.

Species	Nsam				Mendong			
	CDC-LT	HLC	Total	%	CDC-LT	HLC	Total	%
	*N*	*N*			*N*	*N*		
*Aedes* sp.	11	0	11	0.2	5	0	5	0.3
*Culex* sp.	3414	1132	4546	88.1	847	313	1160	58.7
*Mansonia* sp.	3	0	3	0.1	97	36	133	6.7
*An. gambiae* s.l.	212	318	530	10.3	88	197	285	14.4
*An. funestus*	50	20	70	1.4	309	66	375	19.0
*An. ziemanni*	0	0	0	0.0	12	3	15	0.8
*An. marshallii*	0	0	0	0.0	0	3	3	0.2
Total	3690	1470	5160	100	1358	618	1976	100

HLC: human landing catches; CDC-LT: CDC light traps; N: number of mosquitoes.

### Identification of members of the *Anopheles gambiae* complex and *Anopheles funestus* group

A sub-sample of 107 *An. gambiae* s.l. from Nsam and 126 from Mendong randomly selected amongst mosquitoes collected at different periods were further processed to determine sibling species frequency in each district. From the analyses, *An. coluzzii* and *An. gambiae* were present at both sites. *Anopheles coluzzii* presented a frequency varying from 0% to 90.91% in Mendong and from 76.9% to 100% in Nsam (Table 4). In the *An. funestus* group, both *An. funestus* and *An. leesoni* were found at each site. Out of 200 specimens screened by PCR, 46 (23%) were *An. leesoni* and 154 (77%) *An. funestus.* The monthly variation in the frequency of the two species in Nsam and Mendong is presented in Table 4.

**Table 4:** Monthly variation of the composition of species in the *An. gambiae* complex and *An. funestus* group in Nsam and Mendong.

Site	Species	April 17 *N* (%)	June 17 *N* (%)	August 17 *N* (%)	October 17 *N* (%)	December 17 *N* (%)	March 18 *N* (%)	Total *N* (%)
*An. gambiae* s.l.								
Mendong	*An. gambiae*	37 (71.15)	14 (32.56)	3 (20)	2(100)	1 (33.33)	1(9.09)	58 (46)
	*An. coluzzii*	15(28.85)	29 (67.44)	12 (80)	0 (0)	2 (66.67)	10 (90.91)	68 (54)
Nsam	*An. gambiae*	6 (23.08)	2 (6.67)	3 (14.29)	0 (0)	2 (18.18)	0 (0)	13 (12.1)
	*An. coluzzii*	20 (76.92)	28 (93.33)	18 (85.71)	8 (100)	9 (81.82)	11 (100)	94 (87.9)
*An. funestus* s.l.								
Mendong	*An. funestus*	90 (86.54)	12 (70.59)	2 (40)	3 (100)	2 (50)	3 (33.33)	112 (78.9)
	*An. leesoni*	14 (13.46)	5 (29.41)	3 (60)	0 (0)	2 (50)	6 (66.67)	30 (21.1)
Nsam	*An. funestus*	7 (100)	12 (85.71)	7(87.5)	7(63.64)	7(58.33)	2 (33.33)	42 (72.4)
	*An. leesoni*	0 (0)	2 (14.29)	1 (12.5)	4 (33.36)	5 (41.67)	4 (66.67)	16 (27.6)

### Dynamics of anopheline species collected using human landing catches

**Figure 2:**
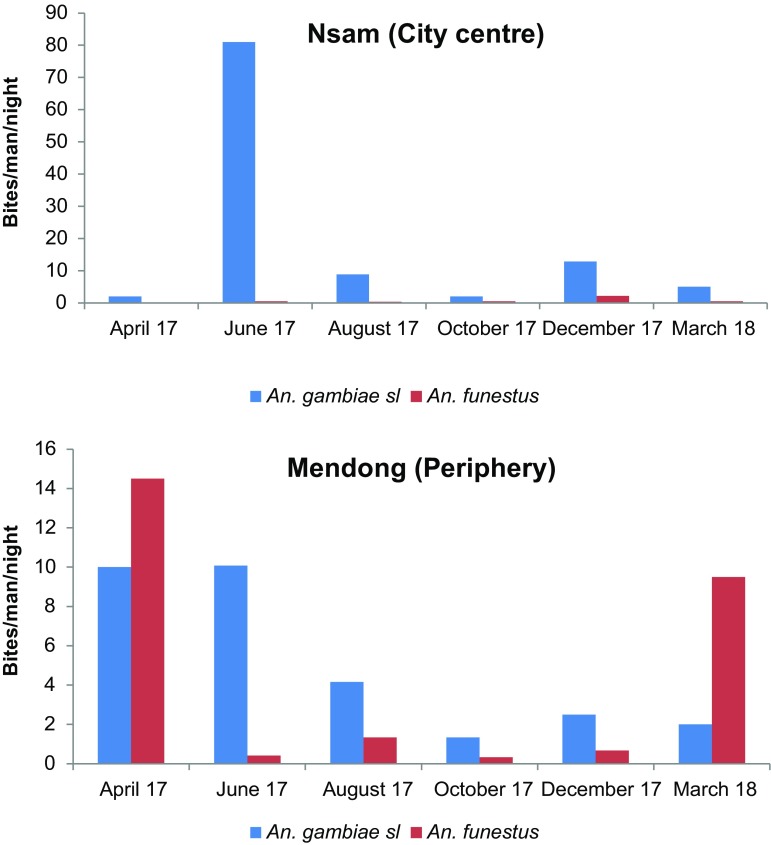
Monthly variation of biting densities of *An. gambiae* s.l. and *An. funestus* collected using HLC in Mendong and Nsam from April 2017 to March 2018.

In Mendong, *An. gambiae* s.l. and *An. funestus* were caught throughout the study period. The *An. gambiae* s.l. human biting rate (HBR) varied from 1.33 to 10.08 bites/man/night (b/m/n), while that of *An. funestus* varied from 0.33 to 14.5 b/m/n. The peak of bites for *An. gambiae* was recorded in June 2017, and the lowest in October 2017, while the highest for *An. funestus* was recorded in April 2017 and the lowest in October 2017 (Fig. 2).

In Nsam, *An. gambiae* s.l. was collected throughout the study period using human landing catches, with the highest biting rate (81 b/m/n) recorded in June 2017. *Anopheles funestus* was less prevalent in the district; its highest biting rate was recorded in December 2017 (2.17 b/m/n).

### Dynamics of anopheline species collected using CDC light traps

**Figure 3:**
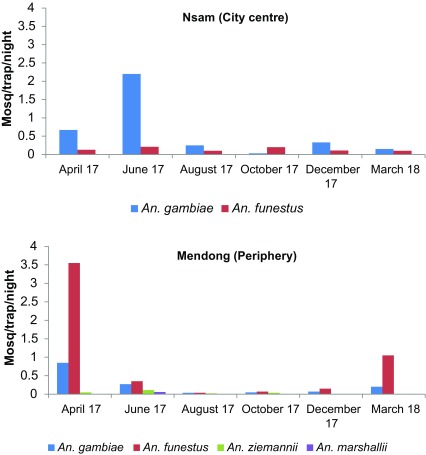
Monthly variation of anopheline densities collected using CDC-LT in Mendong and Nsam from April 2017 to March 2018.

In Mendong, *An. gambiae* densities collected using CDC-LTs varied from 0.04 in August to 0.85 mosquitoes/trap/night in April. Concerning *An. funestus*, its densities varied from 0.04 in August 2017 to 3.55 mosquitoes/trap/night in April 2017. In Nsam, the average density of *An. gambiae* s.l. varied from 0.03 in October 2017 to 2.2 mosquitoes/trap/night in June 2017; however, the density of *An. funestus* varied from 0.1 in August 2017 to 0.21 mosquito/trap/night in June 2017 (Fig. 3).

The Pearson correlation coefficient, used to assess the relationship between CDC-LT and HLC, indicated no significant correlation in the sampling efficiency between CDC-LT and HLC when all mosquitoes were considered in Mendong (*r* = 0.47, *p* = 0.09) whereas a significant correlation was found in Nsam (*r* = 0.67, *p* = 0.03).

### Night biting cycle of anophelines collected with HLC

In Nsam, *An. gambiae* s.l. was recorded as biting all night long. However, there was a peak of bites occurring between 2 am and 3 am indoors (0.75 b/m/h) and between 2 am and 4 am (1.45 b/m/h) outdoors. *Anopheles funestus* was also recorded biting predominantly outdoors during the second part of the night.

**Figure 4:**
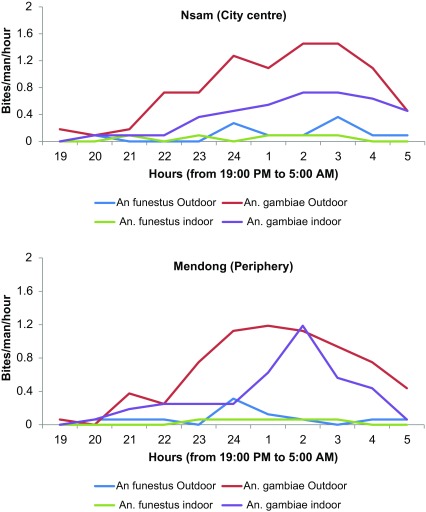
Night biting cycle of *An. gambiae* s.l. and *An. funestus* in Mendong and Nsam.

In Mendong, *Anopheles gambiae* was predominant outdoors, particularly during the second part of the night. Its peak of biting was recorded between midnight and 1 am outdoors (1.19 b/m/h) and 2 am and 3 am indoors (1.19 b/m/h). *Anopheles funestus* biting densities increased from 0 b/m/h to 0.6 b/m/h between midnight and 1 am, and then decreased during the second part of the night (Fig. 4).

### Sporozoite infection rate

Out of 1020 mosquitoes processed using ELISA, 39 were found to be infected: 25 collected using CDC-LTs, distributed as follows: 10 *An. gambiae* s.l. (6 out of 210 at Nsam and 4 out of 84 at Mendong) and 15 *An. funestus* (1 out of 44 at Nsam and 14 out of 296 at Mendong). Similarly, 14 infected mosquitoes were collected by HLC and this included 10 *An. gambiae* s.l. (1 out of 157 at Nsam and 9 out of 145 at Mendong) and 4 *An. funestus* (1 out of 20 at Nsam and 3 out of 64 at Mendong) (Table 5). Two *An. leesoni* specimens within the *An. funestus* group were recorded as infected in Mendong. Of the 20 *An. gambiae* s.l. recorded infected, 10 were *An. coluzzii* (four in Mendong and six in Nsam) and 10 *An. gambiae* (nine in Mendong and one in Nsam). Most infection cases were due to *Plasmodium falciparum* (88%), and the remaining cases (12%) were infections due to *Plasmodium ovale*, *P. malariae*, or *P. vivax*. Infected mosquitoes were detected almost every month at both sites. The infection rate was significantly different between the two sites (*p* < 0.015).

**Table 5:** *Plasmodium falciparum* infection rate of mosquitoes collected using HLCs and CDC-LTs in Nsam and Mendong.

Species	Mendong						Nsam					
	HLC			CDC-LT			HLC			CDC-LT		
	*N*	Infected	IR (95% CI)	*N*	Infected	IR (95% CI)	*N*	Infected	IR (95% CI)	*N*	Infected	IR (95% CI)
*An. gambiae* s.l.	145	9	0.06 (0.03–0.12)	84	4	0.05 (0.01–0.12)	157	1	0.01 (0–0.03)	210	6	0.03 (0.01–0.06)
*An. funestus* s.l.	64	3	0.05 (0.01–0.14)	296	14	0.05 (0.03–0.08)	20	1	0.05 (0–0.28)	44	1	0.02 (0–0.13)
Total	209	12	0.06 (0.03–0.1)	380	18	0.05 (0.03–0.07)	177	2	0.011 (0–0.04)	254	7	0.03 (0.01–0.06)
Overall*	589	30	0.05 (0.03–0.07)				431	9	0.02 (0.01–0.04)			

* Sum of CDC-LT and HLC per site; CDC-LT: CDC light traps; HLCs: human landing catches; IR: infection rate; 95% CI: 95% confidence interval.

### Entomological inoculation rate (EIR)

**Figure 5:**
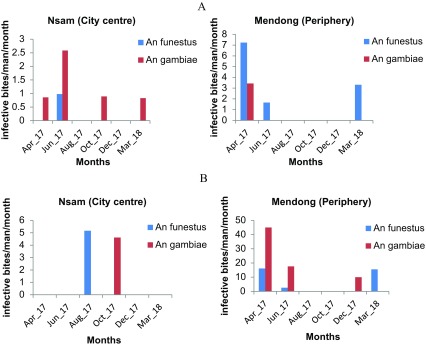
Malaria transmission pattern in Mendong and Nsam from April 2017 to March 2018: (A) monthly EIR using CDC light traps; (B) monthly variation of standard EIR using human landing catches.

Both collections from CDC-LTs and human landing collections were used for EIR estimation. The CDC-LT entomological inoculation rate was estimated at 15.64 infected mosquito bites/man/year (ib/m/yr) at Mendong, and 6.14 ib/m/yr at Nsam. The entomological inoculation rate calculated using HLC was 106.83 ib/m/yr at Mendong and 9.78 ib/m/yr at Nsam (Table 6). Transmission was not recorded during the months of August, October, and December in Mendong, and in August and December at Nsam. Infected mosquitoes collected with CDC-LTs were more regularly observed in Nsam than in Mendong (Fig. 5A and B). The contribution of both *An. funestus* and *An. gambiae* to the annual entomological inoculation rate according to the different collection methods in each study site is presented in Table 6.

**Table 6:** Estimation of the entomological inoculation rate (EIR) using CDC light traps or human landing catches in Nsam and Mendong.

CDC	Mendong		Nsam	
	Annual EIR*	%	Annual EIR*	%
*An. gambiae* s.l.	3.43	21.93	5.16	84.05
*An. funestus* s.l.	12.21	78.07	0.98	15.95
Total	15.64	100	6.14	100
HLC				
*An. gambiae* s.l.	72.59	67.95	4.61	47.18
*An. funestus* s.l.	34.24	32.05	5.17	52.82
Total	106.83	100	9.78	100

* Infective bites/man/year.

### Susceptibility of *An. gambiae* and *An. funestus* to DDT, permethrin, and deltamethrin

A total of 829 *An. gambiae* and 200 *An. funestus* females were exposed to permethrin, deltamethrin, and DDT. *Anopheles funestus* collected at Mendong were resistant to deltamethrin, permethrin and DDT [33.33% (95% CI [21.6–49.2]), 65% (95% CI [48.3–86.4]) and 76% (95% CI [53.4–104.3]) mortality rate, respectively]. Concerning *An. gambiae* females, they were resistant to DDT, deltamethrin, and permethrin at both sites with a mortality rate varying from 0% to 62.5% (Table 7). Of the 60 *An. gambiae* s.l. screened to detect the presence of the *kdr* allele, 49 were detected as carrying the west African *kdr* allele L1014F, whereas three were detected as carrying the east African *kdr* allele L1014S. *Kdr* allele frequency in the population was estimated at 44%.

**Table 7:** Mortality rates for *An. gambiae* s.l. and *An. funestus* field populations after exposure to 4% DDT, 0.75% permethrin, and 0.05% deltamethrin in Mendong and Nsam.

Site	Insecticide	Species					
		*An. gambiae* s.l.			*An. funestus*		
		*N*	Mortality (%)	95% CI	*N*	Mortality (%)	95% CI
Mendong	0.05% deltamethrin	236	62.5	40.3–60.7	75	33.33	21.6–49.2
	0.75% permethrin	193	13.89	42.2–60.8	75	65	48.3–86.4
	4% DDT	100	2	0.2–7.2	50	76	53.8–104.3
Nsam	0.05% deltamethrin	100	3	0.62–8.8			
	0.75% permethrin	100	0	0–3.7			
	4% DDT	100	0	0–3.7			

DDT: dichlorodiphenyltrichloroethane; N: number of mosquitoes tested; 95% CI: 95% confidence interval.

## Discussion

The study objective was to assess the implication of *An. funestus* in malaria transmission in the city of Yaoundé by comparing malaria transmission patterns between an urban and a peri-urban district of the city. High malaria transmission carried by both *An. funestus* and *An. gambiae* s.l. was recorded. The involvement of *An. funestus* in malaria transmission alongside *An. gambiae* contrasted with previous records [23, 46], as well as with the findings from the city of Douala where transmission is mainly sustained by *An. gambiae* s.l. [6, 48]. *Anopheles funestus* has rarely been reported to transmit malaria in urban settings [34, 50]. The typical breeding habitats of *An. funestus* are permanent or semi-permanent water collections with emergent vegetation [26]. Its presence in the city of Yaoundé may result from the presence of marshland covered with emerging vegetation along the Mfoundi and Mefou rivers [21]. *Anopheles funestus* is widely distributed across the country [3] and in most rural settings where it has been reported, it may sustain very high levels of malaria transmission [2, 7, 16, 61]. It was also found to bite predominantly indoors, whereas most *An. gambiae* s.l. bites were recorded outdoors. Both species were recorded to bite frequently during the second part of the night, with *An. gambiae* s.l. recorded biting extensively even after 5 am. This may improve its capacity to maintain residual malaria transmission since after 5 am, most people are out of their bed nets and active (studying for students, cleaning the house, or preparing for the day).

Increased transmission of malaria due to changes in mosquito biting behaviour has been reported in previous studies [40]. High species diversity was recorded in the district of Mendong with six species collected and just four in Nsam. The diversity of species might result from the high variety of breeding habitats in Mendong compared to Nsam with the presence of puddles, lakes, rivers, and swamps, which could be excellent habitats for a variety of mosquito species [2, 4, 11, 21]. Seasonal fluctuations in the biting densities of *An. funestus,* particularly in Mendong, were recorded with a sharp increase at the onset of the rainy season. The abundance of *An. funestus* during this period could be due to the extension of swamps associated with growing vegetation at their edges, which increased breeding opportunities for *An. funestus*. Similar observations were reported elsewhere [17]. *Anopheles gambiae* s.l. was present all year long at both sites; however, there were high densities recorded during the short rainy season (April–June).

Both *An. gambiae* s.l. and *An. funestus* were resistant to permethrin, deltamethrin, and DDT. These findings were consistent with studies conducted across the country reporting rapid evolution of insecticide resistance in these vector populations [9, 38]. The profile of susceptibility in *An. gambiae* s.l. populations to insecticides was similar between the two districts and may suggest similar selective pressure at both sites. In Cameroon, the rapid evolution of insecticide resistance in vector populations is thought to result from selective pressure exerted by the frequent use of treated nets, sprays, and coils in households and pesticide use in agriculture [13, 45]. Although pesticides are commonly used in urban farming, the limited area used for agriculture in Mendong might decrease the impact of selective pressure due to pesticides. It is still not known whether insecticide resistance intensity is similar between the two sites, and this deserves further investigation. Currently, only overexpression of detoxification genes is known to confer resistance to organochlorine and pyrethroids in *An. funestus* populations in Cameroon [38, 52, 53, 67, 68], whereas both target-site mutations and metabolic mechanisms have been reported in *An. gambiae* s.l. populations from Cameroon [9, 44, 58].

*Anopheles funestus* belongs to a group of nine morphologically similar species [14], which can be identified by molecular assays [15, 32]. Three of these species, *An. funestus, An. rivulorum*, and *An. leesoni*, have been reported in Cameroon [15, 41]. Molecular identification indicated the presence of *An. funestus* and *An. leesoni* in both districts. This is the first time *An. leesoni* has been reported in the city of Yaoundé. Interestingly, two *An. leesoni* were also found to be infected. Although *An. leesoni* is frequently reported across the continent, it is considered to have a limited role in malaria transmission [19, 26]. However, it is becoming important to confirm its role as a vector of malaria through dissection of the salivary glands.

Within members of the *An. gambiae* complex, both *An. gambiae* and *An. coluzzii* were recorded. *Anopheles coluzzii* was the predominant species at both sites, representing 87.85% and 53.97% of the total species in Nsam and Mendong, respectively. These data were in accordance with previous studies reporting a heterogeneous distribution of these species in the city of Yaoundé [28, 59].

A high malaria transmission rate was recorded in both districts and likely suggests an elevated malaria transmission risk in both the centre and the city periphery. According to Robert et al. [54], the annual entomological inoculation rate in sub-Saharan Africa could be as high as seven in city centres, 45.8 in peri-urban areas, and 167.7 in rural areas. Transmission levels recorded during this study in the city of Yaoundé were far above these values as well as those previously recorded [23, 60]. Additionally, such high levels likely point to the negative influence of unplanned urbanisation on malaria transmission and are consistent with studies conducted in other major sub-Saharan African cities [6, 30, 34, 37, 42, 43, 48, 50, 54, 69].

## Conclusion

The present study highlights challenges affecting malaria control in the urban environment and confirms the important epidemiological role played by *An. funestus* in the urban environment. In the case of Yaoundé, where vectors display high pyrethroid resistance, different species take part in malaria transmission, and hotspot areas are well identified. The implementation of an integrated control approach combining larvicidal or environmental management by draining urban swamps, with improvements in urban planning, and promotion of the use of treated nets could be indicated for the control and elimination of malaria vectors in Yaoundé.
